# Supplementary material for: Development and application of a tri-allelic PCR assay for screening Vgsc-L1014F kdr mutations associated with pyrethroid and organochlorine resistance in the mosquito Culex quinquefasciatus
Source: Parasit Vectors. 2019 May 14;12:232. doi: 10.1186/s13071-019-3490-z (PMC6518802; doi:10.1186/s13071-019-3490-z)
Supplement: Supplementary file 1 — Additional file 1: Figure S1. Multiple sequence alignment of the partial fragment of the Vgsc gene across Culex pipiens pipiens, Culex p. quinquefasciatus and Culex p. pallens. Figure S2. Neighbor-joining tree of the partial fragment of the Vgsc gene. [file 13071_2019_3490_MOESM1_ESM.pdf]

Forward

```
C.quinq/KR061930.1/1-305       1 GTGGGCGACGTGTCCTGCATTCCGTTCTTCTTGGCCACCGTAGTGATAGGA 51
C.quinq/KR061940.1/1-307       1 GTGGGCGACGTGTCCTGCATTCCGTTCTTCTTGGCCACCGTAGTGATAGGA 51
C.quinq/KR061970.1/1-306       1 GTGGGCGACGTGTCCTGCATTCCGTTCTTCTTGGCCACCGTAGTGATAGGA 51
C.quinq/KM377241.1/1-307       1 GTGGGCGACGTGTCCTGCATTCCGTTCTTCTTGGCCACCGTAGTGATAGGA 51
C.quinq/KM377242.1/1-307       1 GTGGGCGACGTGTCCTGCATTCCGTTCTTCTTGGCCACCGTAGTGATAGGA 51
C.quinq/EF658661.1/1-307       1 GTGGGCGACGTGTCCTGCATTCCGTTCTTCTTGGCCACCGTAGTGATAGGA 51
C.quinq/EF658660.1/1-305       1 GTGGGCGACGTGTCCTGCATTCCGTTCTTCTTGGCCACCGTAGTGATAGGA 51
C.quinq/EF658659.1/1-305       1 GTGGGCGACGTGTCCTGCATTCCGTTCTTCTTGGCCACCGTAGTGATAGGA 51
C.quinq/DQ497226.1/1-306       1 GTGGGCGACGTGTCCTGCATTCCGTTCTTCTTGGCCACCGTAGTGATAGGA 51
C.quinq/DQ497227.1/1-306       1 GTGGGCGACGTGTCCTGCATTCCGTTCTTCTTGGCCACCGTAGTGATAGGA 51
C.pallens/GU198930.1/1-307     1 GTGGGCGACGTGTCCTGCATTCCGTTCTTCTTGGCCACCGTAGTGATAGGA 51
C.pallens/HQ540623.1/1-307     1 GTGGGCGACGTGTCCTGCATTCCGTTCTTCTTGGCCACCGTAGTGATAGGA 51
C.pipiens/DQ497213.1/1-307     1 GTGGGCGACGTGTCCTGCATTCCGTTCTTCTTGGCCACCGTAGTGATAGGA 51
C.pipiens/DQ497230.1/1-306     1 GTGGGCGACGTGTCCTGCATTCCGTTCTTCTTGGCCACCGTAGTGATAGGA 51
C.pipiens/DQ497228.1/1-306     1 GTGGGCGACGTGTCCTGCATTCCGTTCTTCTTGGCCACCGTAGTGATAGGA 51
C.pipiens/DQ497214.1/1-306     1 GTGGGCGACGTGTCCTGCATTCCGTTCTTCTTGGCCACCGTAGTGATGGGA 51

C.quinq/KR061930.1/1-305      52 AATTTAGTCGTGAGTATTCCAGCGTGAAGTCTTAGCGATTGATCTAGTGTG 102
C.quinq/KR061940.1/1-307      52 AATTTTGTCGTGAGTATTCCAGCGTGAAGTCTTAGCGATTGATCTAGTGTG 102
C.quinq/KR061970.1/1-306      52 AATTTCGTCGTGAGTATTCCAGCGTGAAGTCTTCGTGATTGATCTAGTGTG 102
C.quinq/KM377241.1/1-307      52 AATTTTGTCGTGAGTATTCCAGCGTGAAGTCTTAGCGATTGATCTAGTGTG 102
C.quinq/KM377242.1/1-307      52 AATTTAGTCGTGAGTATTCCAGCGTGAAGTCTTAGCGATTGATCTAGTGTG 102
C.quinq/EF658661.1/1-307      52 AATTTTGTCGTGAGTATTCCAGCGTGAAGTCTTAGCGATTGATCTAGTGTG 102
C.quinq/EF658660.1/1-305      52 AATTTAGTCGTGAGTATTCCAGCGTGAAGTCTTAGCGATTGATCTAGTGTG 102
C.quinq/EF658659.1/1-305      52 AATTTAGTCGTGAGTATTCCAGCGTGAAGTCTTAGCGATTGATCTAGTGTG 102
C.quinq/DQ497226.1/1-306      52 AATTTTGTCGTGAGTATTCCAGCGTGAAGTCTTAGCGATTGATCTAGTGTG 102
C.quinq/DQ497227.1/1-306      52 AATTCAGTCGTGAGTATTCCAGCGTGAAGTCTTAGCGATTGATCTAGTGTG 102
C.pallens/GU198930.1/1-307    52 AATTTTGTCGTGAGTATTCCAGCGTGAAGTCTTAGCGATTGATCTAGTGTG 102
C.pallens/HQ540623.1/1-307    52 AATTTTGTCGTGAGTATTCCAGCGTGAAGTCTTAGCGATTGATCTAGTGTG 102
C.pipiens/DQ497213.1/1-307    52 AATTTTGTCGTGAGTATTCCAGCGTGAAGTCTTAGCGATTGATCTAGTGTG 102
C.pipiens/DQ497230.1/1-306    52 AATTTAGTCGTGAGTATTCCAGCGTGAAGTCTTAGCGATTGATCTAGTGTG 102
C.pipiens/DQ497228.1/1-306    52 AATTTAGTCGTGAGTATTCCAGCGTGAAGTCTTCGTGATTGATCTAGTGTG 102
C.pipiens/DQ497214.1/1-306    52 AATTTTGTCGTGAGTATTCCAGCGTGAAGTCTTCGTGATTGATCTAGTGTG 102

C.quinq/KR061930.1/1-305     103 CGCGCTAGAGCTGTCAAAACATCGCCAACAGCATGCAAGAAAAGGTGGGAA 153
C.quinq/KR061940.1/1-307     103 CGCGCTAGAGCTGTCAAAACATCGCCAACAGCATGCAAGAAAAGGTGGGAA 153
C.quinq/KR061970.1/1-306     103 CGCATTAGAGCTGTCAAAACATCGCCAACAGCATGCAAGAAAAGGTGGGAA 153
C.quinq/KM377241.1/1-307     103 CGCGCTAGAGCTGTCAAAACATCGCCAACAGCATGCAAGAAAAGGTGGGAA 153
C.quinq/KM377242.1/1-307     103 CGCGCTAGAGCTGTCAAAACATCGCCAACAGCATGCAAGAAAAGGTGGGAA 153
C.quinq/EF658661.1/1-307     103 CGCGCTAGAGCTGTCAAAACATCGCCAACAGCATGCAAGAAAAGGTGGGAA 153
```

C.quinq/KR061930.1/1-305	103	CGCGCTAGAGCTGTCAAAACATCGCCAACAGCATGCAAGAAAAGGTGGGAA	153
C.quinq/KR061940.1/1-307	103	CGCGCTAGAGCTGTCAAAACATCGCCAACAGCATGCAAGAAAAGGTGGGAA	153
C.quinq/KR061970.1/1-306	103	CGCATTAGAGCTGTCAAAACATCGCCAACAGCATGCAAGAAAAGGTGGGAA	153
C.quinq/KM377241.1/1-307	103	CGCGCTAGAGCTGTCAAAACATCGCCAACAGCATGCAAGAAAAGGTGGGAA	153
C.quinq/KM377242.1/1-307	103	CGCGCTAGAGCTGTCAAAACATCGCCAACAGCATGCAAGAAAAGGTGGGAA	153
C.quinq/EF658661.1/1-307	103	CGCGCTAGAGCTGTCAAAACATCGCCAACAGCATGCAAGAAAAGGTGGGAA	153
C.quinq/EF658660.1/1-305	103	CGCGCTAGAGCTGTCAAAACATCGCCAACAGCATGCAAGAAAAGGTGGGAA	153
C.quinq/EF658659.1/1-305	103	CGCGCTAGAGCTGTCAAAACATCGCCAACAGCATGCAAGAAAAGGTGGGAA	153
C.quinq/DQ497226.1/1-306	103	CGCGCTAGAGCTGTCAAAACATCGCCAACAGCATGCAAGAAAAGGTGGGAA	153
C.quinq/DQ497227.1/1-306	103	CGCGCTAGAGCTGTCAAAACATCGCCAACAGCATGCAAGAAAAGGTGGGAA	153
C.pallens/GU198930.1/1-307	103	CGCGCTAGAGCTGTCAAAACATCGCCAACAGCATGCAAGAAAAGGTGGGAA	153
C.pallens/HQ540623.1/1-307	103	CGCGCTAGAGCTGTCAAAACATCGCCAACAGCATGCAAGAAAAGGTGGGAA	153
C.pipiens/DQ497213.1/1-307	103	CGCGCTGGAGCTGTCAAAACATCGCCAACAGCATGCAAGAAAAGGTGGGAA	153
C.pipiens/DQ497230.1/1-306	103	CGCGCTAGAGCTGTCAAAACATCGCCAACAGCATGCAAGAAAAGGTGGGAA	153
C.pipiens/DQ497228.1/1-306	103	CGCATTAGAGCTGTCAAAACATCGCCAACAGCATGCAAGAAAAGGTGGGAA	153
C.pipiens/DQ497214.1/1-306	103	CGCATTAGAGCTGTCAAAACATCGCCAACAGCATGCAAGAAAAGGTGGGAA	153

Reverse ←

C.quinq/KR061930.1/1-305	154	CGAAAAACTTTAAGGTCACATTTGTACCTTT	GATGTAAACAAACAGTTCAT	204
C.quinq/KR061940.1/1-307	154	CGAAAAACTTTAAGGTCACATTTGTACCTTT	GATGTAAACAAACAGTTCAT	204
C.quinq/KR061970.1/1-306	154	CGAAAAACTTTAAGGTCACATTTGTACCTTT	GATGTAAACAAACAGTTCAT	204
C.quinq/KM377241.1/1-307	154	CGAAAAACTTTAAGGTCACATTTGTACCTTT	GATGTAAACAAACAGTTCAT	204
C.quinq/KM377242.1/1-307	154	CGAAAAACTTTAAGGTCACATTTGTACCTTT	GATGTAAACAAACAGTTCAT	204
C.quinq/EF658661.1/1-307	154	CGAAAAACTTTAAGGTCACATTTGTACCTTT	GATGTAAACAAACAGTTCAT	204
C.quinq/EF658660.1/1-305	154	CGAAAAACTTTAAGGTCACATTTGTACCTTT	GATGTAAACAAACAGTTCAT	204
C.quinq/EF658659.1/1-305	154	CGAAAAACTTTAAGGTCACATTTGTACCTTT	GATGTAAACAAACAGTTCAT	204
C.quinq/DQ497226.1/1-306	154	CGAAAAACTTTAAGGTCACATTTGTACCTTT	GATGTAAACAAACAGTTCAT	204
C.quinq/DQ497227.1/1-306	154	CGAAAAACTTTAAGGTCACATTTGTACCTTT	GATGTAAACAAACAGTTCAT	204
C.pallens/GU198930.1/1-307	154	CGAAAAACTTTAAGGTCACATTTGTACCTTT	GATGTAAACAAACAGTTCAT	204
C.pallens/HQ540623.1/1-307	154	CGAAAAACTTTAAGGTCACATTTGTACCTTT	GATGTAAACAAACAGTTCAT	204
C.pipiens/DQ497213.1/1-307	154	CGAAAAACTTTAAGGTCACATTTGTACCTTT	GATGTAAACAAACAGTTCAT	204
C.pipiens/DQ497230.1/1-306	154	CGAAAAACTTTAAGGTCACATTTGTACCTTT	GATGTAAACAAACAGTTCAT	204
C.pipiens/DQ497228.1/1-306	154	CGAAAAACTTTAAGGTCACATTTGTACCTTT	GATGTAAACAAACAGTTCAT	204
C.pipiens/DQ497214.1/1-306	154	CGAAAAACTTTAAGGTCACATTTGTACCTTT	GATGTAAACAAACAGTTCAT	204

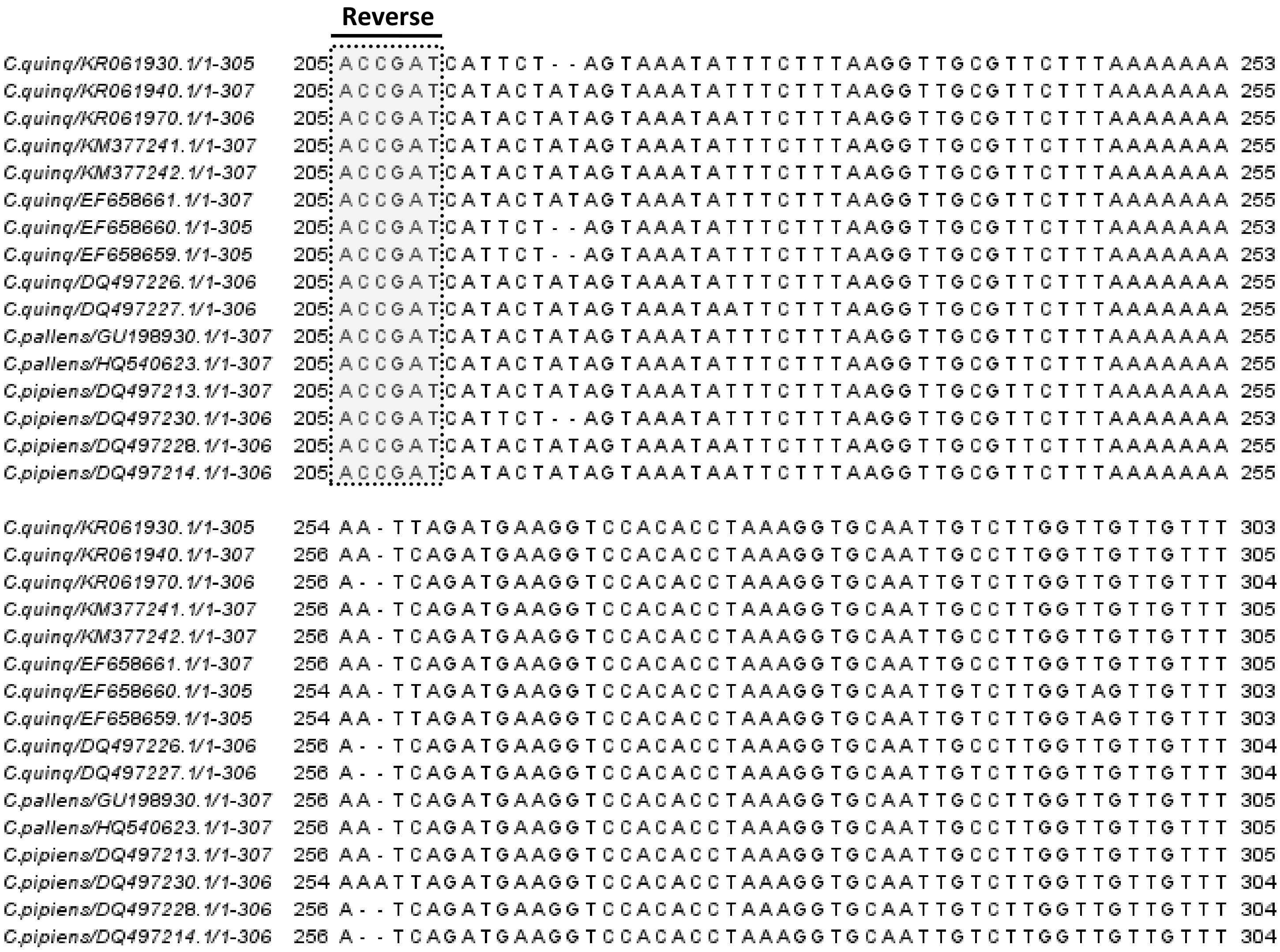

Figure S1. Multiple sequences alignment of the partial fragment of the *Vgsc* gene across *Culex pipiens pipiens*, *Culex p. quinquefasciatus* and *Culex p. pallens*. Alignment was performed using MEGA software (1). Grey shaded boxes correspond to forward and reverse primer sequences. The red line in the forward primer highlights the position of the *Vgsc*-1014 mutation.

1. Tamura K, Peterson D, Peterson N, Stecher G, Nei M, Kumar S. MEGA5: molecular evolutionary genetics analysis using maximum likelihood, evolutionary distance, and maximum parsimony methods. Molecular Biology and Evolution. 2011;28(10):2731-9.

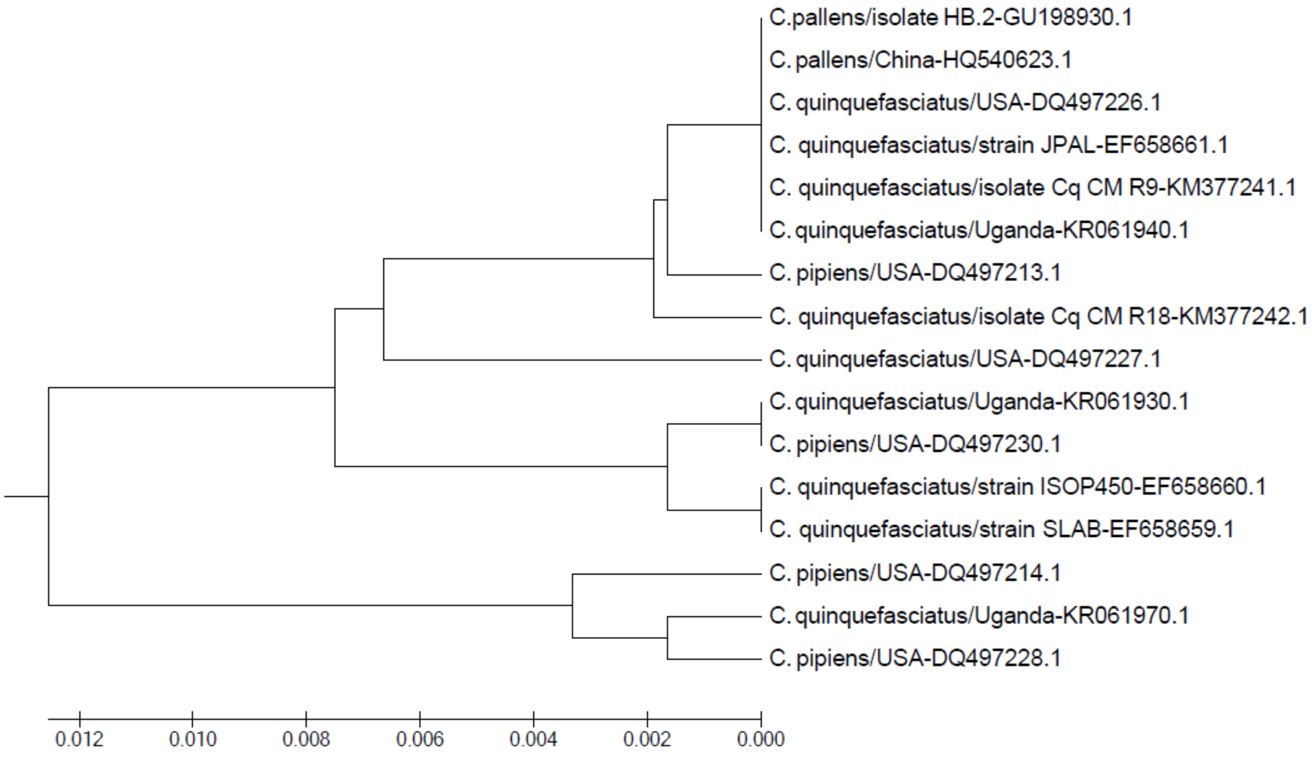

Figure S2. Neighbor-joining tree of the partial fragment of the *Vgsc* gene. Branch names are composed of; *Culex* species/ geographic origin or strain name, and GenBank accession number.
