# Supplementary material for: Development and application of a tri-allelic PCR assay for screening Vgsc-L1014F kdr mutations associated with pyrethroid and organochlorine resistance in the mosquito Culex quinquefasciatus
Source: Parasit Vectors. 2019 May 14;12:232. doi: 10.1186/s13071-019-3490-z (PMC6518802; doi:10.1186/s13071-019-3490-z)
Supplement: Supplementary file 2 — Additional file 2: Table S1. Allelic and genotypic frequency of Vgsc-1014F in relation to mosquito survival phenotype by deltamethrin, lambda-cyhalothrin or DDT in C. quinquefasciatus from Tororo, Uganda. [file 13071_2019_3490_MOESM2_ESM.pdf]

Table S1. Allelic and genotypic frequency of *Vgsc*-1014F in relation to mosquito survival phenotype by deltamethrin, lambda-cyhalothrin or DDT in *C. quinquefasciatus* from Tororo, Uganda.

Insecticide	N	Survivorship	Phenotype	Genotype						*Vgsc*-1014F allele freq[a]	*Vgsc*-1014F genotype freq[b]
				AA**	CC	TT	AC	AT	CT		
Deltamethrin	24	66.66% *(44.66-83.66)	Alive	3	7		5		1	65.6%	81.25
			Dead	3	2		1	1	1		
lambda-cyhalothrin	30	93.33% *(77.9-99.13)	Alive	4	1		8	7	8	58.8%	85.71
			Dead	2							
DDT	30	90% *(73.5-97.9)	Alive	8	5	1	9	1	3	51.8%	70.37
			Dead	3							

* 95% confidence intervals

** Wild-type genotype

[a;] frequency within alive

[b;] frequency within alive excluding wild-type homozygous
